# Vertical Stacking Statistics of Multi-facies Object-Based Models

**DOI:** 10.1007/s11004-023-10046-0

**Published:** 2023-02-14

**Authors:** Tom Manzocchi, Deirdre A. Walsh

**Affiliations:** iCRAG and Fault Analysis Group, UCD School of Earth Sciences, University College Dublin, Dublin 4, Ireland

**Keywords:** Object-based model, Amalgamation ratio, Facies proportion, Compression-based model, Compression algorithm

## Abstract

Equations describing facies proportions and amalgamation ratios are derived for randomly placed objects belonging to two or three foreground facies embedded in a background facies, as a function of the volume fractions and object thicknesses of independent facies models combined in a stratigraphically meaningful order. The equations are validated using one-dimensional continuum models. Evaluation of the equations reveals a simple relationship between an effective facies proportion and an effective amalgamation ratio, both measured as a function only of the facies in question and the background facies. This relationship provides a firm analytical basis for applying the compression algorithm to multi-facies object-based models. A set of two-dimensional cross-sectional models illustrates the approach, which allows models to be generated with realistic object stacking characteristics defined independently for each facies in a multi-facies object-based model.

## Introduction

The objectives of this work are specific and straightforward: to establish analytical expressions for certain statistics of one-dimensional vertical samples through object-based models. The motivation for deriving precise expressions is so the stacking properties of modelled systems can be conditioned to match those of natural systems, as part of broader endeavours to build more realistic subsurface models. Classification of sedimentary deposits into distinct facies is standard practice in both academic sedimentological studies and practical subsurface modelling, and the present work is focused on systems comprising one, two or three foreground facies embedded in a background facies. The foreground facies are composed of objects that represent discrete depositional elements. The geometries and stacking characteristics of elements within a particular facies are assumed to be similar to each other, but are different between the facies. Hence, for example, a deep-water system might contain discrete lobate and channelised elements modelled as separate facies, and the geometries and stacking characteristics of the elements will vary depending on whether the modelled volume is contained within the channel, channel-lobe transition zone or lobe of the large, fan-scale deposit (e.g. Cullis et al. 2018; Fryer and Jobe 2019).

The statistics of interest to this study are facies proportions and object amalgamation ratios. Facies proportions (defined as the fractional volumes of the model occupied by each facies) are a common measure usually specified as an input property of object-based models. The amalgamation ratio is a measure of the connectivity of the objects and is usually an unconstrained output property of object-based models. It is typically measured at the bed scale, where it is defined as the number of bed bases in contact with an underlying bed (as opposed to shale) as a proportion of the total number of bed bases present. This definition follows Chapin et al. (1994) and was used by Stephen et al. (2001), Manzocchi et al. (2007) and Romans et al. (2009). More recently, Jobe et al. (2021) and Stanbrook and Bentley (2022) discussed practicalities of acquiring the ratio from one-dimensional data. A quite different definition of amalgamation ratio is also sometimes used in the literature (e.g. Mueller et al. 2017; Pyles et al. 2019). This alternative definition was introduced because of the difficulty of identifying individual amalgamated surfaces and is based on the thicknesses of amalgamated and non-amalgamated intervals, rather than on counts of bed bases. It is unfortunate that the same name is used for the two ratios, because although they will usually be strongly correlated, they are not the same. Considerations by Manzocchi et al. (2020, their Fig. 4) show how the former ratio might be calculated from the latter, based on an assumed bed thickness. Other measures of vertical stacking which are closely related to the amalgamation ratio have also been discussed (e.g. Funk et al. 2012; Jackson et al. 2019).

Bed-scale amalgamation ratios have been particularly well characterised in the slope and basin floor submarine channel systems of the Magallanes Basin in Chile (e.g. Romans et al. 2009; Jobe et al. 2010; Macauley and Hubbard 2013). In general, channelised systems have much larger amalgamation ratios than basin floor fan systems (Mattern 2002) for which amalgamation ratios have been measured by Manzocchi et al. (2007), Marini et al. (2015) and Pyles et al. (2019). Amalgamation ratios have also been examined at multiple, larger hierarchical levels in both lobate and channelised deep-water systems (e.g. Zhang et al. 2015; Manzocchi et al. 2020; Soni et al. 2020) with the general trend that larger scales of depositional elements tend to be more poorly amalgamated than smaller ones.

**Fig. 1:**
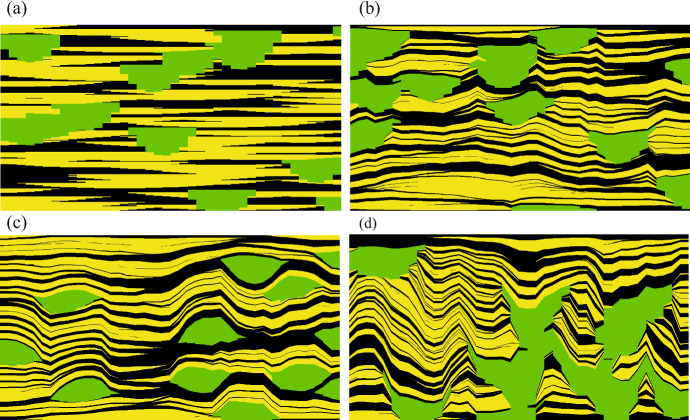
Cross sections through models with the same facies proportions and object shapes for two foreground facies (yellow sheets and green channels) in a black background facies. See text for discussion

We have shown elsewhere that natural depositional systems generally have lower amalgamation ratios than are obtained in object- or pixel-based models (e.g. Walsh and Manzocchi 2021a; Manzocchi et al. in press a) and have proposed an approach (compression-based modelling) in which a geometrical transformation to a facies model results in stacking properties that cannot be obtained in conventional models but which are representative of natural systems (e.g. Manzocchi et al. 2007, 2020, in press b; Walsh and Manzocchi 2021a, b). Examples generated using cross-sectional object-based models consisting of two foreground facies with sheet- and channel-shaped objects are shown in Fig. 1. Figure 1a shows a model in which the compression algorithm has not been applied, and hence the facies amalgamation ratios are unconstrained. The channels are more erosive than the sheets because they are thicker, but there are places in the model where sheets erode into the top of channels. The object stacking pattern in the model (Fig. 1a) is created by the stratigraphically appropriate facies preservation rule included in the object-based modelling algorithm and will be similar for all object-based model realisations created with these facies proportions and shapes. The other three models (Fig. 1b to d) have the same facies proportions and shapes, but in these cases the compression algorithm has been used to produce models with different staking characteristics, resulting in more poorly amalgamated sheets with unconstrained channels (Fig. 1b), more poorly amalgamated sheets and channels (Fig. 1c) and more poorly amalgamated sheets but more highly amalgamated channels (Fig. 1d). These two-dimensional models demonstrate the impact that object amalgamation ratios can have on the spatial distributions of the facies, but also highlight the most notable geometrical artefact of the compression algorithm which is the imposition of dip caused by lateral facies transitions (Manzocchi et al. 2020). This artefact is not addressed in this paper which focuses on one-dimensional model characteristics.

The transformation within the compression method applied to object-based models relies on a statement of the target values of facies proportions and amalgamation ratios, and expressions defining how these properties are related to each other in the initial object-based model prior to the transformation. This paper is concerned with establishing these expressions for multi-facies models. The approach taken is to derive algebraic expressions for the stacking characteristics of idealised one-dimensional systems and to validate these expressions using numerical models. The following section defines in detail the system examined and the stacking characteristics of interest, and introduces the one-dimensional object-based modelling code used to illustrate the problem and validate the results. Sections 3, 4 and 5 develop analytical expressions for systems containing one, two and three foreground facies. The results are verified at the end of their respective sections, and Sect. 6 discusses the results and illustrates how they might be used in practice. Appendix 1 lists, and explains the logic behind, the abbreviations and symbols used throughout the paper.

## Definition of the Problem

The one-dimensional system considered in this paper consists of up to three foreground facies (F1, F2 and F3) embedded in a background facies (F0) over a total model height (*H*). For most of the derivations in this paper, *H* is assumed to be 1 and therefore does not generally appear in the equations. In certain places, however, it is useful to refer to *H* explicitly. Throughout the paper, X is used as a general facies indicator to denote any of F1, F2 or F3. Objects of facies FX have constant thickness ($T_X$), and the facies are ordered and named to satisfy the condition $T_1 \le T_2 \le T_3$. The background facies (F0) is not modelled as discrete objects, and therefore is not associated with a particular object thickness value. The models considered in this paper are non-hierarchical, and the results could apply to any level in a depositional hierarchy. Hence, although the objects are referred to as beds throughout this paper for convenience, they could just as well represent lobes, channels or other depositional elements. The assumption of constant thickness ($T_X$) for objects belonging to each facies simplifies the derivations in the paper. Implications of the assumption for models with a single foreground facies are discussed by Manzocchi et al. (in press b), who show that models with broader thickness distributions have a marginally lower amalgamation ratio for the same net:gross ratio.

**Fig. 2:**
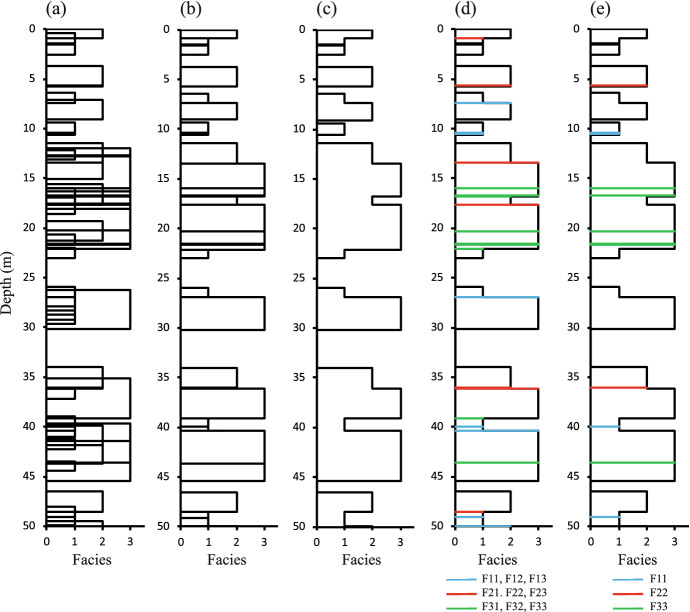
Example one-dimensional three-facies model showing **a** the initial bed model, **b** the stacked bed model and **c** the facies model. In **d** the facies model shows all amalgamation surfaces, and in **e** it shows only amalgamations with beds of the same facies. The model has $V_1$ = $V_2$ = $V_3$ = 0.4, $T_1$ = 1m, $T_2$ = 2m, $T_3$ = 4m and *H* = 50m

The code used to generate the numerical models includes many of the same assumptions as the analytical solutions derived later, and therefore a model realisation is used to illustrate the problem being addressed (Fig. 2). Initially, each facies and each bed is treated independently. Beds are assumed to be positioned randomly and are permitted to overlap with each other and with the top and bottom edges of the volume of interest (Fig. 2a). Placement of beds for each facies continues until the facies occupies a specific volume fraction ($V_X$) of the total model height. The random placement of beds implies that the probability that each facies is present at any location in the initial bed model is simply equal to $V_X$ and is independent of location in the column. This assumption is consistent with continuum Boolean modelling approaches (Shante and Kirkpatrick 1971; Baker et al. 2002) and is central to the analytical derivation of object stacking statistics described in this work.

The initial model (Fig. 2a) can contain multiple beds of the same or different facies at any position which must be rationalised to produce a stratigraphically meaningful stacked model. The stacked bed model (Fig. 2b) has only one facies present at any location and is created from the initial model by assuming that the preservation of overlapping beds is governed by Steno’s law of superposition (Steno 1669). Hence, beds which have their tops higher in the sequence are assumed to be stratigraphically younger and to erode into stratigraphically older beds which have their upper surface further down the sequence. The beds in the numerical model are generated in a continuum, so there is never any ambiguity as to which bed is younger, and only one sequence of beds can be generated from the initial model which satisfies the law of superposition. The stacked model contains fewer beds overall than the initial model, since some are entirely eroded out of the sequence. Many of the remaining beds are partially eroded by an overlying bed, and therefore the beds in the stacked model have variable thicknesses.

The facies model (Fig. 2c) is the same as the stacked model but does not contain any information about the contacts between different beds. The most important properties of the facies model are the facies proportions ($P_X$), which is one of the fundamental properties defined as input in practical facies modelling exercises. It is clear from Fig. 2c that in this example, $P_3> P_2> P_1$, despite the fact that the same volume fractions were used to generate the initial model ($V_1= V_2= V_3= 0.4$, Fig. 2a). This is because where two facies overlap in the initial model, the thicker bed is more likely to have a higher top than the thinner bed, and therefore is more likely to be preserved given a stratigraphically sensible stacking order. Establishing a precise definition of $P_X$ as a function of $V_X$ and $T_X$ is one of the objectives of the analyses in this paper.

The version of the model shown in Fig. 2d is the same as the facies model (Fig. 2c) except that all contacts between beds are coloured: these contacts are termed amalgamations and are labelled using the convention that FXY represents an amalgamation surface defined by an FX bed above an FY bed. Using this scheme, a non-amalgamated FX bed base that overlies F0 is termed FX0. The choice of colour used depends on the facies above the contact. Hence, blue lines represent amalgamated bases of F1 beds, red lines amalgamated bases of F2 beds, and green lines amalgamated bases of F3 beds. The black horizontal lines (Fig. 2d) represent unamalgamated bed edges where either the top of the bed is overlain by shale (F0) or the base of the bed overlies shale. Quantifying the proportions of amalgamated bed bases is an important measure of the overall three-dimensional connectivity of the facies, and if only one facies is present, the amalgamation ratio (AR) is equal to the facies proportion if all beds are the same thickness (Manzocchi et al. 2007). Establishing an expression for AR in the presence of multiple facies is the second objective of the analyses below.

Two different definitions of facies-specific AR are used in this paper: total AR and effective AR ($AR_{TX}$ and $AR_{EX}$, respectively). Both measures were used also by Manzocchi et al. (in press b) and are defined as follows, using the term $n_{XY}$ to signify the number of beds of facies X that amalgamate with a bed of facies Y, such that

1 $$\begin{aligned} AR_{TX}= & {} \frac{n_{X1} + n_{X2}+ n_{X3}}{n_{X0}+ n_{X1} + n_{X2}+ n_{X3}}; \end{aligned}$$

2 $$\begin{aligned} AR_{EX}= & {} \frac{n_{XX}}{n_{X0}+ n_{XX}}. \end{aligned}$$

The difference between $AR_{TX}$ and $AR_{EX}$ is shown by the difference between Fig. 2d, e: the former includes all amalgamations at the bases of beds of the facies of interest, while the latter considers only amalgamations with beds of the same facies.

The amalgamation ratios used in this paper are related to, but distinct from, the embedded facies transition probabilities often used to characterise stratigraphic logs (Krumbein and Dacey 1969). Amalgamation ratios refer to the bases of individual beds and are therefore based on the information contained in the stacked bed model (Fig. 2b). Facies transition probabilities, however, refer only to facies distributions and therefore are based on the subset of information contained in the facies model (Fig. 2c). Amalgamation between beds of the same facies is not a characteristic recorded in the facies model, and $n_{XX}$ is undefined in the facies transition probability approach. The association between the two sets of parameters can be appreciated by stating the embedded facies transition probability ($p_{XY}$) with the terminology used in this paper. In this way, the probability that facies FX transitions downwards to facies FY is given by

3 $$\begin{aligned} p_{XY} = \frac{n_{XY}}{n_{X0}+ n_{X1} + n_{X2}+ n_{X3}-n_{XX}}. \end{aligned}$$

This equation contains $n_{XX}$ twice: one explicitly, and once as one of the other terms in the denominator. These cancel out, so $p_{XY}$ does not depend on $n_{XX}$ (the equation has been written in this form so it can be generalised).

Facies transition probability matrices lie at the heart of the Markov chain approach for modelling stratigraphic sections (Krumbein and Dacey 1969; Carle and Fogg 1997, 2020; Li et al. 2019), but there is no fundamental reason why amalgamation ratios (or the associated bed transition probability matrix) should not be used instead. In the Markov chain approach, a volume is populated with facies sequentially and in a specific direction, resulting in non-random sequences built as a function of input facies transition probabilities. However, this is not the approach taken in this paper which instead assumes the random bed placement method described above and calculates the resultant amalgamation ratios from the resultant sequence. The reason for taking this approach is because it is closely allied to the object-based modelling approach often used to build two- and three-dimensional facies models (Fig. 1). As discussed in the Introduction, if the amalgamation ratios of object-based models are known as a function of model input parameters, a geometrical transformation can be used to create models with specific, non-random amalgamation ratios representative of natural geological systems.

## Stacking Properties of Single-Facies Models

The properties of models containing only one foreground facies (F1) are simple. It is obvious that $P_1 = V_1$ since no other objects are present. Less obvious is the fact that $AR_{E1} = V_1$. The reason for this was explained by Walsh and Manzocchi (2021a) as follows: if all beds are of equal thickness, then it is impossible for a higher bed to erode the base of a lower bed. Therefore, all bed bases are preserved in the stacked bed model. Since the beds are located randomly, so too are the bed bases. Hence, there is a probability of exactly $V_1$ that each bed is positioned on top of (and therefore is amalgamated with) a lower bed, and a probability of exactly $(1-V_1)$ that it is not amalgamated. Therefore, if there are $n_1$ beds altogether, the expected numbers of F11 and F10 bed bases are

4 $$\begin{aligned} n_{11}=n_1V_1 \end{aligned}$$

and

5 $$\begin{aligned} n_{10}=n_1(1-V_1). \end{aligned}$$

These can be combined using either Eq. 1 or 2, since the different versions of AR are the same when only one facies is present. This yields

6 $$\begin{aligned} AR_1=V_1. \end{aligned}$$

Bed stacking can be considered in a bit more detail to derive the distribution of bed thicknesses in the stacked model. This is necessary for the solutions involving two or three foreground facies derived in the following sections. Shante and Kirkpatrick (1971) showed that the expected total number of beds in the system ($n_1$) is given by

7 $$\begin{aligned} n_1=-\frac{H}{T_1}ln(1-V_1). \end{aligned}$$

This is because, if a bed is added to a system that already has a facies proportion $P_1$, then the facies proportion will increase, on average, to $P_1+\frac{T_1}{H}(1-P_1)$. Expressing this as a differential equation gives

8 $$\begin{aligned} dn_1=\frac{HdP_1}{T_1(1-P_1)}, \end{aligned}$$

which, when integrated, gives Eq. 7.

The bed bases are located at random, so it must be the case that the distances between bed bases follow a negative exponential distribution. When these distances are less than the original thickness of the beds ($T_1$), the distance must represent the thicknesses of beds which have been partially eroded and are capped by an amalgamation surface (i.e. $t_1<T_1$). It is impossible for the bed thickness to exceed $T_1$, and therefore, distances greater than $T_1$ represent beds that are preserved in their entirety ($t_1=T_1$), plus an overlying region of F0. Hence, the distribution of preserved F1 bed thicknesses is

9 $$\begin{aligned} n_{t1}= & {} n_1^2e^{-n_1t_1}dt_1 \text { for } 0<t_1<T_1 \nonumber \\ \text { and } n_{t1}= & {} n_1(1-V_1) \text { for } t_1=T_1, \end{aligned}$$

where $n_{t1}$ is the number of beds of thickness in the range $t_1$ to $t_1+dt_1$.

**Fig. 3:**
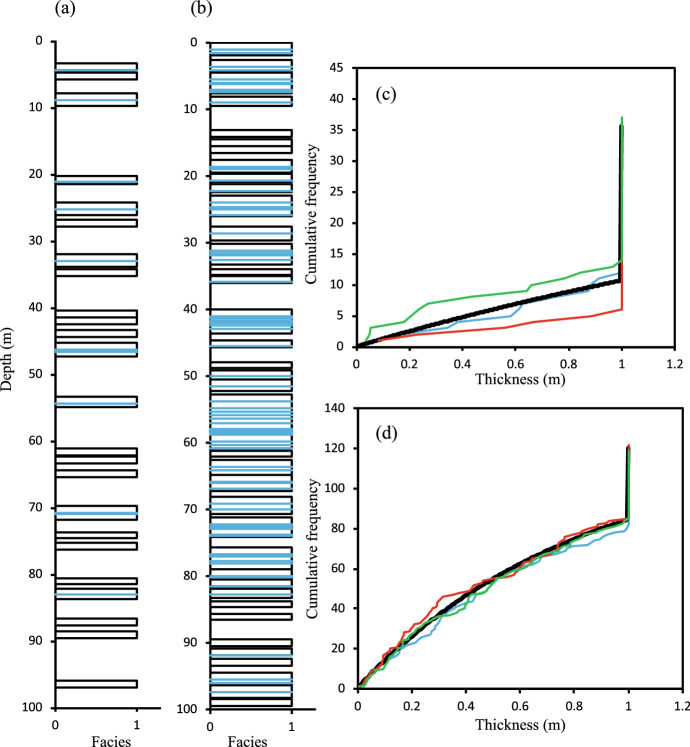
Stacked bed models of systems with $T_1$ = 1m, *H* = 100m and **a** $V_1$ = 0.3, **b** $V_1$ = 0.7. Blue lines show amalgamation surfaces. **c**, **d** Analytically derived cumulative distribution (black) and cumulative distribution of three realisations (coloured lines) of resultant bed thickness (t) for the models shown in a and b, respectively

The ideal distribution (Eq. 9) is compared to that obtained in three realisations of two systems (Fig. 3). Differences between realisations reflect the small size of the systems considered in this illustration, but they clearly follow the expected distribution.

## Stacking Properties of Models Containing Two Facies

### Introduction

When two foreground facies (F1 and F2) potentially with different thicknesses are present ($T_2 \ge T_1$), there is the potential to entirely erode out F1 beds and, as discussed in the Introduction, we expect that $P_2 \ge P_1$ even if $V_2 = V_1$, because thicker beds are more likely to be erosive. Calculation of facies proportions and amalgamation ratios are therefore much more complicated than when only one facies is present.

**Fig. 4:**
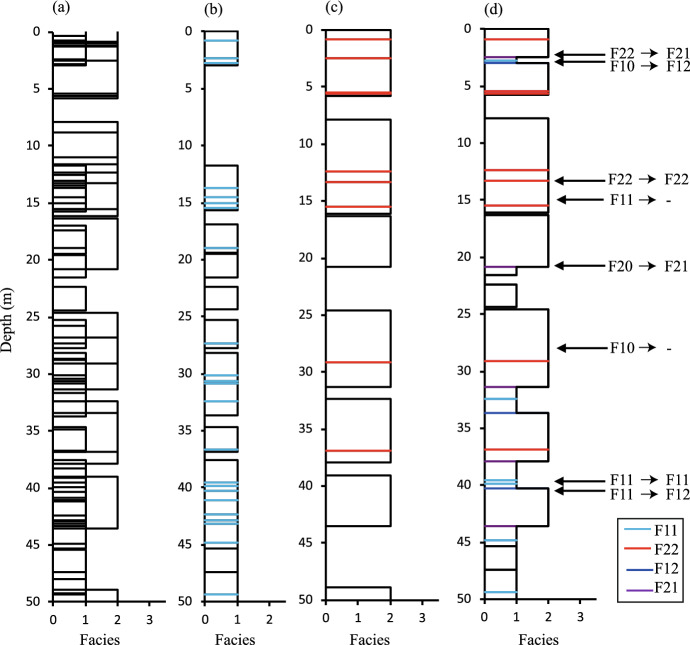
Two-facies model with $T_1$ = 2m, $T_2$ = 4.5m, $V_1$ = 0.7, $V_2$ = 0.7 and *H* = 50m. **a** Initial bed model of both facies. Single-facies stacked models of **b** facies 1 and **c** facies 2 including coloured F11 and F22 surfaces. **d** Combined stack models using different colours for each type of amalgamation surface. The labelled contacts in **d** show examples of all possible outcomes of all possible surface types. See Table 1 and text for discussion

The initial model (Fig. 4a) can be converted to independent single-facies stacked models in which $P_1 = V_1$ and $P_2= V_2$ (Fig. 4b, c). Since the location of beds is entirely random, there is a probability equal to $V_1V_2$ that any position is occupied by beds belonging to both F1 and F2. The particular bed retained at these locations in the combined stacked model (Fig. 4d) is a function of the locations of the two bed tops and will be calculated later. For now, it is simply expressed as a facies preservation probability ($p_{1\mid 12}$) that F1 rather than F2 is preserved at locations where both are present. It follows, therefore, that the facies proportions in the combined stacked model (Fig. 4d) are

10 $$\begin{aligned} P_1=V_1-V_1V_2(1-p_{1\mid 12}) \end{aligned}$$

and

11 $$\begin{aligned} P_2=V_2-V_1V_2p_{1\mid 12}. \end{aligned}$$

The amalgamation ratios are calculated as a function of different types of bed bases (Eqs. 1, 2). An F1 bed base in a single-facies stacked model can be one of two types, F11 or F10, with the quantities of each given by Eqs. 4 and 5. In positions where a bed base in a single-facies model overlies a bed from the other facies, the surfaces can either be preserved, changed to a different type of surface, or eroded out of the system altogether. The possible outcomes for these surfaces are summarised in Table 1. The outcome probabilities in Table 1 are expressed in the form $p_{XXXY\mid XY}$, which means that an FXX surface from a single-facies model is preserved as an FXY surface in the two-facies model when the surface is directly above a position occupied by beds of both facies X and Y in the independent single-facies stacked models. In these expressions, a hyphen (−) is used to denote erosion, so $p_{X0-\mid Y}$ refers to the probability that an FX0 surface is eroded out of the system where an FY bed overlaps it. Further details of the precise logic applied to defining the symbols are given in Appendix 1.

**Table 1:** Possible outcomes of bed bases in areas of F1 and F2 overlap when the independent single-facies stacked models are merged

Surface in single-facies	Resultant surface in the	Probability of this	Location of an
model	two-facies model	outcome ($p_{OB}$)	example (Fig. 4)
F11	F11	$p_{1111 \mid 12}$	39 m
	F12	$p_{1112 \mid 12}$	40 m
	Eroded out	$p_{11- \mid 12}$	15 m
F10	F12	$p_{1012 \mid 2}$	2.5 m
	Eroded out	$p_{10- \mid 2}$	24 m
F22	F22	$p_{2222 \mid 12}$	14 m
	F21	$p_{2221 \mid 12}$	2 m
F20	F21	$p_{2021 \mid 1}$	21 m

These surface outcome probabilities ($p_{OB}$; Table 1) apply only where the bed base coincides with a position occupied by the other facies. This coincidence is defined by a surface overlap probability ($p_{OA}$) which is equal to the volume fraction of the other facies. If the other facies is not present at this locality, the base must be preserved. So, for example, an F11 base cannot be transformed into anything else where there is nothing else to interfere with it. Hence, $p_{1111 \mid 1}=1$ and $p_{2222 \mid 2}=1$.

Table 1 omits terms for $p_{22- \mid 1}$ and $p_{20- \mid 1}$, which would both be associated with an F1 bed eroding out an F2 bed base. This is impossible (i.e. has a probability of zero), since the facies are defined to satisfy the condition that $T_1 \le T_2$, and a bed cannot erode down to the base of a thicker (or equal thickness) bed.

The surface outcome probabilities listed in Table 1 (which will be calculated in Sect. 4.3) can be combined with the number of bed bases present in the single-facies models (Eqs. 4 and 5) and the surface overlap probabilities ($p_{OA}$) to calculate the number of bed bases of different types ($n_{XY}$) which are required to calculate the amalgamation ratios (Eqs. 1 to 3). For example,

12 $$\begin{aligned} n_{11}=n_1V_1((1-V_2)+V_2p_{1111\mid 12}), \end{aligned}$$

where the first term ($n_1V_1$) is the number of F11 surfaces in the single-facies model (Fig. 4b, Eq. 5), the second term ($1-V_2$) is the proportion of these surfaces that do not coincide with F2 in the other single-facies model (Fig. 4c) and therefore cannot be altered, and the third term ($V_2p_{1111 \mid 12}$) gives the probable proportion of these contacts that could be altered but which are not.

Performing a similar calculation for the other bed contact types, we obtain

13 $$\begin{aligned} n_{12}= & {} n_1V_1V_2p_{1112\mid 12}+n_1(1-V_1)V_2p_{1012\mid 2}, \end{aligned}$$

14 $$\begin{aligned} n_{10}= & {} n_1(1-V_1)(1-V_2), \end{aligned}$$

15 $$\begin{aligned} n_{21}= & {} n_2V_1V_2p_{2221\mid 12}+n_2(1-V_2)V_1p_{2021\mid 1}, \end{aligned}$$

16 $$\begin{aligned} n_{22}= & {} n_2V_2(1-V_2)+V_1p_{2222\mid 12}, \end{aligned}$$

and

17 $$\begin{aligned} n_{20}=n_2(1-V_1)(1-V_2). \end{aligned}$$

The discussion above and Eqs. 1, 2 and 10 to 17 show how the facies proportions and amalgamation ratios can be calculated in a two-facies model. The equations contain various probabilities that define the likelihood that different facies, or types of bed bases, are preserved when the stack model (Fig. 4d) is created from the single-facies models (Fig. 4b, c). The following two sub-sections address these probabilities within the context of the facies proportions and amalgamation ratio calculations.

### Facies Proportions

**Fig. 5:**
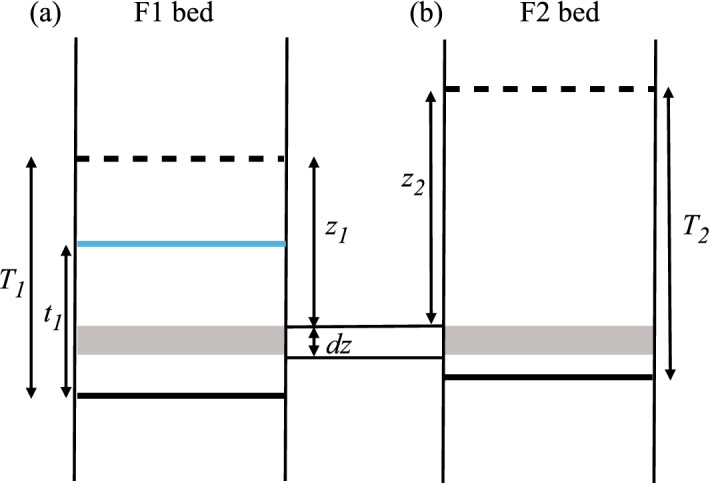
**a** An F1 bed of original thickness $T_1$ is partially eroded to leave a thickness $t_1$. The solid black line represents the bed base, the dashed black line the original bed top, and the blue line shows the amalgamation surface (and hence the resultant top of the bed). **b** An F2 bed is also present at the location of interest (the shaded region *dz*). Whether F1 or F2 eventually occupies this locality depends on whether or not $z_1 > z_2$

The key to the facies proportion calculations (Eqs. 10 and 11) is the probability term $p_{1 \mid 12}$, which expresses the probability that F1 is preserved where both F1 and F2 are present. To solve $p_{1 \mid 12}$, it is necessary to consider location as a function of distance below the top of a bed irrespective of whether or not this bed top has been eroded. This requires consideration of three length terms. The first two are $T_X$ and $t_X$, which are the thickness of the initial input beds and of the resultant, potentially eroded, beds in the single-facies stacked models, respectively. The third length term ($z_X$) is defined as a distance below the original bed top (Fig. 5). The approach taken is to calculate the total thickness of preserved facies X material present at a particular distance (i.e. between $z_X$ and $z_X+dz$; Fig. 5) below bed tops in the single-facies stacked models (e.g. Fig. 4b, c). This thickness (which is termed $Lz_X$ and which increases with increasing $z_X$ since higher portions of beds are more likely to be eroded) is then used to derive the probabilities required.

For every bed of preserved thickness $t_X \ge T_X-z_X$ , there is a thickness *dz* that contributes to $Lz_X$ (Fig. 5). Hence,

18 $$\begin{aligned} Lz_X=\int ^{t_X=T_X}_{t_X=T_X-z_X} n_{tX}dz, \end{aligned}$$

where $n_{tX}$ is the number of beds of thickness $t_X$ which is given by Eq. 7. Replacing this in Eq. 18 and solving, we obtain

19 $$\begin{aligned} Lz_X= n_X(1-V_X)e^{n_Xz_X}{\textrm{d}}z. \end{aligned}$$

Figure 5 illustrates a situation in which both an F1 and an F2 bed are present within the distance $z_X$ to $z_X+dz$. If $z_1 > z_2$, F1 will be preserved and F2 eroded, and if $z_1 < z_2$ (the case illustrated in Fig. 5), F2 will be preserved and F2 eroded. The case $z_1 = z_2$ does not need to be considered, since the beds are considered within a continuum in which it is vanishingly probable that $z_1 = z_2$.

For a particular value of $z_1$, the probability that F1 is preserved is given by

20 $$\begin{aligned} p_{1\mid 12\mid z_1}= \frac{\int ^{z_2=z_1}_{0} L_{z2}}{\int ^{z_2=T_2}_{0} L_{z2}}. \end{aligned}$$

Replacing Eq. 19 into Eq. 20, we have

21 $$\begin{aligned} p_{1\mid 12\mid z_1}= \frac{(1-V_2)}{V_2}(e^{n_2z_1}-1). \end{aligned}$$

To get the full probability $p_{1 \mid 12}$ for all values of $z_1$ requires integration over the full possible range of $z_1$. Hence,

22 $$\begin{aligned} p_{1\mid 12}= \frac{\int ^{z_1=T_1}_{0} P_{1\mid 12\mid z_1}L_{z1}}{\int ^{z_1=T_1}_{0} L_{z1}}, \end{aligned}$$

which ultimately gives

23 $$\begin{aligned} p_{1\mid 12}= \frac{n_1(1-V_1)(1-V_2)}{V_1V_2}\Bigg ( \frac{e^{(n_1+n_2)T_1}-1}{n_1+n_2}- \frac{e^{n_1T_1}-1}{n_1} \Bigg ). \end{aligned}$$

Applying this in Eqs. 10 and 11 gives the facies proportions $P_1$ and $P_2$ as a function of expected number of beds ($n_1$ and $n_2$, given by Eq. 7) and the system input parameters $T_1$, $T_2$, $V_1$ and $V_2$. Note that $n_1$ and $n_2$ are themselves functions of $T_1$, $T_2$, $V_1$ and $V_2$, and so could have been eliminated as terms in this expression. However, we have found it more convenient here and elsewhere to keep $n_1$ and $n_2$ as separate terms in the equations.

### Amalgamation Ratios

**Fig. 6:**
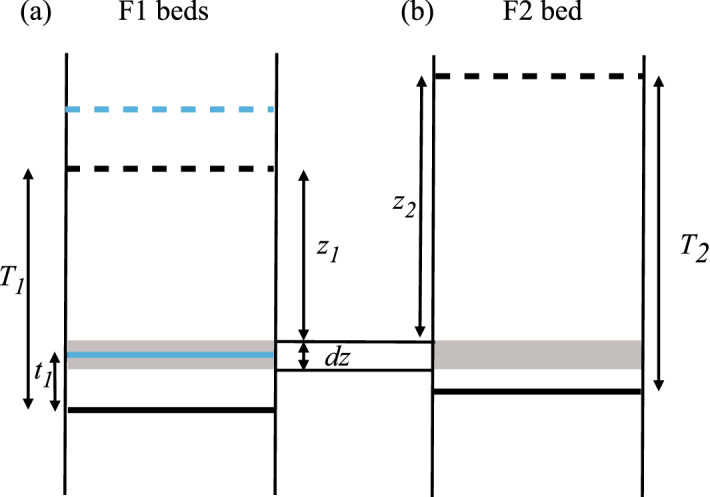
**a** An F1 bed of original thickness $T_1$ is partially eroded to leave a thickness $t_1$. The dashed and solid blue lines represent the original top and base of the overlying eroding bed; the dashed and solid black lines are the same for the lower, eroded bed. **b** An F2 bed also occupies the same locality as the amalgamation surface (within the shaded region *dz*). Whether the F11 surface (the solid blue line) is eroded out, remains an F11 surface or is transformed into a F12 surface depends on the locations of the two F1 beds and the F2 bed

**Fig. 7:**
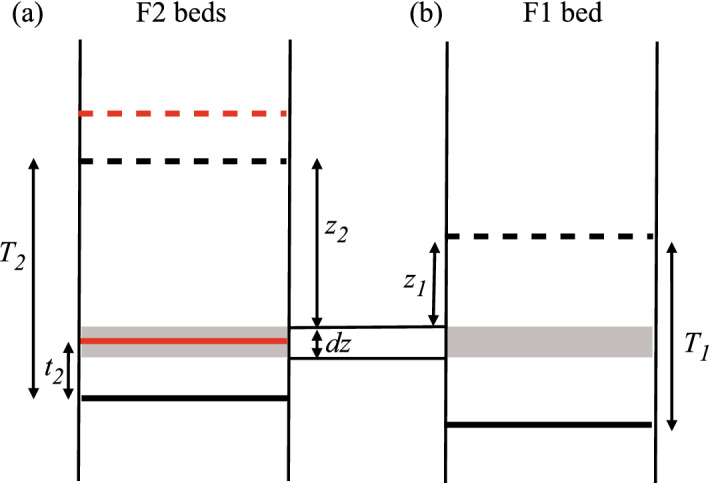
**a** An F2 bed of original thickness $T_2$ is partially eroded to leave a thickness $t_2$. The dashed and solid red lines represents the original top and base of the overlying eroding bed, and the dashed and solid black lines the same for the lower, eroded bed. **b** An F1 bed also occupies the same locality as the amalgamation surface (within the shaded region dz). Whether the F22 surface (the solid red line) remains an F22 surface or is transformed into a F21 surface depends on whether the original top of the F2 bed is above or below the original top of the F1 bed. The surface cannot be eroded out, since the top of the upper F2 bed must be above the top of the F1 bed

Calculation of the amalgamation ratios requires consideration of different probabilities for four different types of surface in the single-facies models (Table 1). These are considered in turn below, in each case by first considering a constant value of $z_1$ or $z_2$ (Figs. 6, 7) and then integrating over the full range of possible values of $z_1$ or $z_2$ to provide the final probabilities required.

#### Outcomes for F11 Surfaces

Figure 6 considers the possible outcome of an F11 surface (Fig. 6a) when its position coincides with an F2 bed (Fig. 6b). There are three possible outcomes. If $z_2>T_1$, it will be eroded out of the system because all of the upper F1 bed, including its amalgamated base, will be replaced by the F2 bed. If $T_1> z_2>z_1$, it will be transformed into an F12 surface because the top of the lower F1 bed, but not the base of the upper F1 bed, will be replaced by F2. If $z_1 > z_2$, it remains an F11 surface since the lower F1 bed erodes out this portion of the F2 bed. These three cases are defined by the probabilities $p_{11- \mid 12}$, $p_{1112 \mid 12}$ and $p_{1111 \mid 12}$, respectively (Table 1). The first of these does not need to be calculated, as it does not feature in any of the equations used to determine the number of different bed base types (Equations 12-17), but the other two do.

A similar procedure to the one applied to derive $p_{1 \mid 12 \mid z_1}$ (Sect. 4.2) yields

24 $$\begin{aligned} p_{1111\mid 12\mid z_1}= \frac{(1-V_2)}{V_2}(e^{n_2z_1}-1) \end{aligned}$$

and

25 $$\begin{aligned} p_{1112\mid 12\mid z_1}= \frac{(1-V_2)}{V_2}(e^{n_2T_1}-e^{n_2z_1}). \end{aligned}$$

Integrating over the full possible range of $z_1$ values gives the probabilities

26 $$\begin{aligned} p_{1111\mid 12}= \frac{n_1(1-V_1)(1-V_2)}{V_1V_2}\Bigg ( \frac{e^{(n_1+n_2)T_1}-1}{n_1+n_2}- \frac{e^{n_1T_1}-1}{n_1} \Bigg ) \end{aligned}$$

and

27 $$\begin{aligned} p_{1112\mid 12}= \frac{n_1(1-V_1)(1-V_2)}{V_1V_2}\Bigg ( \frac{e^{(n_1+n_2)T_1}-e^{n_2T_1}}{n_1}- \frac{e^{(n_1+n_2)T_1}-1}{n_1+n_2} \Bigg ). \end{aligned}$$

Note that $p_{1111 \mid 12}$ (Eq. 26) is identical to $p_{1 \mid 12}$ (Eq. 23), but $p_{1112 \mid 12}$ is not the same as $p_{2 \mid 12}$ (which was not calculated explicitly in Sect. 4.2 since $p_{2 \mid 12} = 1- p_{1 \mid 12})$. This is because a surface can be eroded entirely out of the system, so there are three possible outcomes for each surface. But one or other facies must be preserved, so there are only two possible outcomes for facies proportions.

#### Outcomes for F10 Surfaces

There are only two possible outcomes for unamalgamated F1 bed bases: they either become F12 surfaces, or they are eroded out of the system. Only the first of these outcomes feature in Eqs. 13 to 17, so only $p_{1012 \mid 2}$ needs to be calculated. Transformation of the bed base to an F12 surface requires $z_2<T_1$. Therefore,

28 $$\begin{aligned} p_{1012\mid 2}= \frac{\int ^{z_2=T_1}_{0} L_{z2}}{\int ^{z_2=T_2}_{0} L_{z2}}, \end{aligned}$$

which gives

29 $$\begin{aligned} p_{1012\mid 2}= \frac{(1-V_2)}{V_2} (e^{n_2T_1}-1). \end{aligned}$$

#### Outcomes for F22 Surfaces

For the F22 surfaces, two probabilities are important ($p_{2222 \mid 12}$ and $p_{2221 \mid 12}$), but only one needs to be calculated since $p_{2222 \mid 12} = (1-p_{2221 \mid 12}$). The criterion differentiating between these possibilities is whether or not $z_1 > z_2$ (Fig. 7). Considering the case where $z_1 > z_2$, for a particular value of $z_2$ we have

30 $$\begin{aligned}{} & {} p_{2221\mid 12\mid z_2}= \frac{(1-V_1)}{V_1} (e^{n_1T_1}-e^{n_1z_2}) \text { if } z_2<T_1 \nonumber \\ \text { and }{} & {} p_{2221\mid 12\mid z_2}=0 \text { if } z_2>T_1. \end{aligned}$$

This integrates across all values of $z_2$ to give

31 $$\begin{aligned} p_{2221\mid 12}= \frac{n_2(1-V_1)(1-V_2)}{V_1V_2}\Bigg ( \frac{e^{(n_1+n_2)T_1}-e^{n_1T_1}}{n_2}- \frac{e^{(n_1+n_2)T_1}-1}{n_1+n_2} \Bigg ). \end{aligned}$$

Additionally, as discussed,

32 $$\begin{aligned} p_{2222\mid 12}=1-p_{2221\mid 12}. \end{aligned}$$

#### Outcomes for F20 Surfaces

F20 bed bases cannot be eroded out of the system. Therefore, the only thing that can happen to them if their position in the F2 facies model (Fig. 4c) coincides with F1 beds in the F1 facies model (Fig. 4b) is that they are transformed into a F21 contact. Therefore,

33 $$\begin{aligned} p_{2021\mid 1}=1. \end{aligned}$$

### Validation of the Two-Facies Results

Results are validated by running a simple experiment consisting of 27 (i.e. $3^3$) one-dimensional continuum models, comprising all possible combinations of three settings of three variables. Variables are $V_1$ and $V_2$, for which settings of [0.15, 0.5, 0.85] are used, and $T_2$ using values of [1.0m, 1.5m, 5.0m]. $T_1$ is kept constant at 1.0m, and model resolution is defined by setting the model height (*H*) to a value consistent with an expectation of at least 100 beds for each facies. Hence, *H* is different in different models and is calculated by taking the model height to be the minimum of $H_1$ and $H_2$, where (following Eq. 7) these facies-specific heights are given by $H_X = - \frac{100T_X}{ln(1-V_X)}$.

**Fig. 8:**
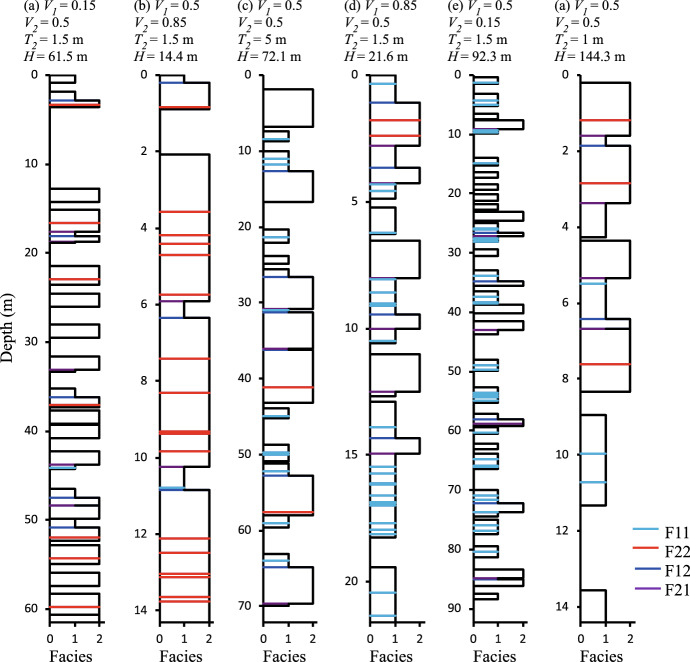
Realisations at one-tenth resolution for six of the 27 systems examined

**Fig. 9:**
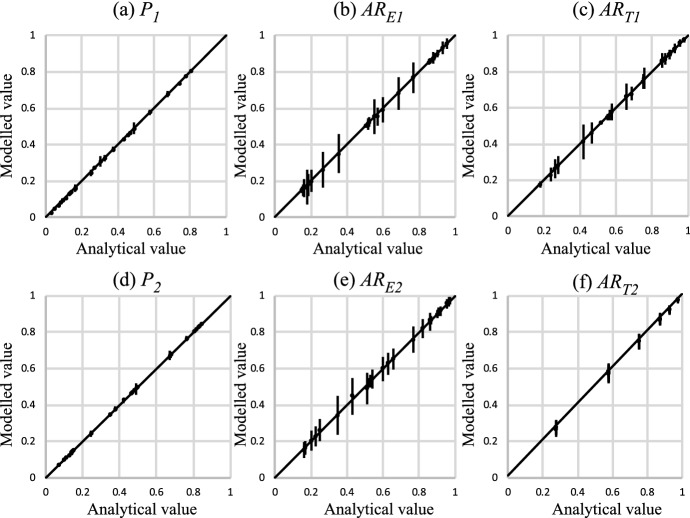
Comparison between the analytically derived facies proportions and amalgamation ratios, and those obtained in the numerical models, for the two facies cases. Error bars represent +/- one standard deviation around the mean value obtained in 100 realisations. See text for discussion

Example models are shown at a scale of *H*/10 in Fig. 8, and results for 100 realisations of all systems in Fig. 9. These graphs show unbiased 1:1 relationships between the analytically derived expected values and modelled values of facies proportions (Fig. 9a, d) and amalgamation ratios (Fig. 9b, c, e, f), and therefore confirm the equations.

## Stacking Properties of Models Containing Three Facies

The solution for three facies follows the same basic considerations as for two facies. The facies are ordered such that $T_1 \le T_2 \le T_3$, and the objective is to derive the facies proportions ($P_X$) and amalgamation ratios ($AR_{TX}$, $AR_{EX}$) as a function of these thicknesses, and of the three input volume fractions $V_1$, $V_2$ and $V_3$.

### Facies Proportions

The probability that all three facies overlie each other at any location is $V_1V_2V_3$, and that two facies (FX and FY) but not the third (FW) are present at any location is $V_XV_Y(1-V_W)$. Hence, the facies proportions in a combined three-facies model (e.g. Fig. 2c) are given by

34 $$\begin{aligned} P_1= & {} V_1-V_1V_2(1-V_3)(1-p_{1 \mid 12})-V_1V_3(1-V_2)(1-p_{1 \mid 13})\nonumber \\ {}{} & {} -V_1V_2V_3(1-p_{1 \mid 123}), \end{aligned}$$

35 $$\begin{aligned} P_2= & {} V_2-V_1V_2(1-V_3)p_{1 \mid 12}-V_2V_3(1-V_1)(1-p_{2 \mid 23})\nonumber \\ {}{} & {} -V_1V_2V_3(1-p_{2 \mid 123}), \end{aligned}$$

and

36 $$\begin{aligned} P_3=V_3-V_1V_3(1-V_2)p_{1 \mid 13}-V_2V_3(1-V_1)p_{2 \mid 23}-V_1V_2V_3(p_{1 \mid 123}+p_{2 \mid 123}).\nonumber \\ \end{aligned}$$

The probability $p_{1 \mid 12}$ has already been calculated (Eq. 23), and $p_{1 \mid 13}$ and $p_{2 \mid 23}$ are derived in exactly the same way. They are

37 $$\begin{aligned} p_{1\mid 13}= \frac{n_1(1-V_1)(1-V_3)}{V_1V_3}\Bigg ( \frac{e^{(n_1+n_3)T_1}-1}{n_1+n_3}- \frac{e^{n_1T_1}-1}{n_1} \Bigg ), \end{aligned}$$

and

38 $$\begin{aligned} p_{2\mid 23}= \frac{n_2(1-V_2)(1-V_3)}{V_2V_3}\Bigg ( \frac{e^{(n_2+n_3)T_2}-1}{n_2+n_3}- \frac{e^{n_2T_2}-1}{n_2} \Bigg ). \end{aligned}$$

The probabilities $p_{1 \mid 123}$ and $p_{2 \mid 123}$ are a little more complicated since the locations of beds of three different facies need to be considered. However, the same basic approach (Sect. 4.2) is applied to give

39 $$\begin{aligned} p_{1\mid 123}= & {} \frac{n_1(1-V_1)(1-V_2)(1-V_3)}{V_1V_2V_3}\Bigg ( \frac{e^{(n_1+n_2+n_3)T_1}-1}{n_1+n_2+n_3}- \frac{e^{(n_1+n_3)T_1}-1}{n_1+n_3} \nonumber \\{} & {} - \frac{e^{(n_1+n_2)T_1}-1}{n_1+n_2}+\frac{e^{n_1T_1}-1}{n_1} \Bigg ), \end{aligned}$$

and

40 $$\begin{aligned} p_{2\mid 123}= & {} \frac{n_2(1-V_2)(1-V_3)}{V_2V_3}\Bigg ( \Bigg ( \frac{(1-V_1)}{V_1} \Bigg ) \Bigg ( \frac{e^{(n_1+n_2+n_3)T_1}-1}{n_1+n_2+n_3}- \frac{e^{(n_1+n_2)T_1}-1}{n_1+n_2} \nonumber \\{} & {} - \frac{e^{(n_2+n_3)T_1}-1}{n_2+n_3}+\frac{e^{n_2T_1}-1}{n_2} \Bigg ) + \frac{e^{(n_2+n_3)T_2}-e^{(n_2+n_3)T_1}}{n_2+n_3}-\frac{e^{n_2T_2}-e^{n_2T_1}}{n_2} \Bigg ).\nonumber \\ \end{aligned}$$

### Bed Base Types

When three rather than two facies are considered, the type and origin of possible bed bases increases significantly. Table 2 lists all possible outcomes of the two types of bed bases present in the stacked single-facies F1 models when they are merged to form the combined three-facies model. Similar tables have been constructed for F2 and F3 bed bases but are not shown since only one table is required to explain the general procedure followed. The probabilities of the different outcomes for the F11 and F10 bed bases are rationalised in Table 2 using two different terms. The first term is the probability that the location of a particular type of bed base in the single-facies model is coincident with the presence of other specific facies in the other single-facies models, and is termed $p_{OA}$. The second term is the probability that a particular surface type results from the interactions of facies present, and is termed $p_{OB}$. There are often several different combinations of situations that can lead to the formation of a particular surface type, and the total number of surfaces is calculated by summing these outcomes. For example, there are four ways of creating F13 surfaces (Table 2), and the total expected number of F13 surfaces is given by

41 $$\begin{aligned} n_{13}= & {} n_1V_1(p_{11 \mid 13}p_{1113 \mid 13}+p_{11 \mid 123}p_{1113 \mid 123})\nonumber \\ {}{} & {} +n_1(1-V_1)(p_{10 \mid 3}p_{1013 \mid 3}+p_{10 \mid 23}p_{1013 \mid 23}). \end{aligned}$$

The shorter terms in this expression ($p_{11 \mid 13}$, $p_{11 \mid 123}$, $p_{10 \mid 3}$ and $p_{10 \mid 23}$) are the overlap probabilities generalised as $p_{OA}$, and the longer terms ($p_{1113 \mid 13}$, $p_{1113 \mid 123}$, $p_{1013 \mid 3}$ and $p_{1013 \mid 23}$) are the outcome probabilities generalised as $p_{OB}$ (Table 2). These two types of probability ($p_{OA}$, and $p_{OB}$) are dealt with in the next two sub-sections.

**Table 2:** Possible outcomes and associated probabilities of surfaces in the F1 single-facies models when it is combined into the three-facies model

Type (and number)	Facies present	Probability	Resultant surface	Probability of this
of bed bases in	directly below	of this	in the	outcome given this
single-facies model	this surface	overlap ($p_{OA}$)	three-facies model	overlap ($p_{OB}$)
F11 ($n_1V_1$)	F1	$p_{11 \mid 1}$	F11	1.0
	F1, F2	$p_{11 \mid 12}$	F11	$p_{1111 \mid 12}$
			F12	$p_{1112 \mid 12}$
			Eroded	$p_{11- \mid 12}$
	F1, F3	$p_{11 \mid 13}$	F11	$p_{1111 \mid 13}$
			F13	$p_{1113 \mid 13}$
			Eroded	$p_{11- \mid 13}$
	F1, F2, F3	$p_{11 \mid 123}$	F11	$p_{1111 \mid 123}$
			F12	$p_{1112 \mid 123}$
			F13	$p_{1113 \mid 123}$
			Eroded	$p_{11- \mid 123}$
F10 ($n_1(1-V_1)$)	None	$p_{10 \mid 0}$	F10	1.0
	F2	$p_{10 \mid 2}$	F12	$p_{1012 \mid 2}$
			Eroded	$p_{10- \mid 2}$
	F3	$p_{10 \mid 3}$	F13	$p_{1013 \mid 3}$
			Eroded	$p_{10- \mid 3}$
	F2,F3	$p_{10 \mid 23}$	F12	$p_{1012 \mid 23}$
			F13	$p_{1013 \mid 23}$
			Eroded	$p_{10- \mid 23}$

### Number of Bed Bases and Overlap Probabilities

There are two types of bed bases in the single-facies stacked models: bases that overlie another bed of the same facies (FXX) and bases that overlie shale (FX0). The expected numbers of these are given by

42 $$\begin{aligned} n_{XX}= n_XV_X, \end{aligned}$$

and

43 $$\begin{aligned} n_{X0}= n_X(1-V_X), \end{aligned}$$

where $n_X$ (the number of beds) is given by Eq. 7.

The overlap probabilities ($p_{OA}$) define the probability that one of these bed bases in the single-facies model lies in the same position as one or more different facies in the other single-facies models. The are six probabilities for each facies (e.g. Table 2) which are straightforward to calculate and are given by the general equations

44 $$\begin{aligned} p_{XX \mid X}= & {} p_{X0 \mid 0}=(1-V_Y)(1-V_W), \end{aligned}$$

45 $$\begin{aligned} p_{XX \mid XY}= & {} p_{X0 \mid Y}=V_Y(1-V_W), \end{aligned}$$

and

46 $$\begin{aligned} p_{XX \mid XYW}= p_{X0 \mid YW}=V_YV_W, \end{aligned}$$

where X, Y and W refer to the three foreground facies, and 0 to the background one.

### Surface Outcome Probabilities

Table 2 lists all possible surface outcome probabilities ($p_{OB}$) associated with F1 beds, but not all need to be calculated, since some are associated with outcomes that are not included in the amalgamation ratio calculations (e.g. $p_{10- \mid 23}$, associated with eroding out a bed based), and others can be combined to simplify the equations. For example, the term $p_{1113 \mid 123}$ (present in Eq. 41 to derive $n_{13}$) can be combined with $p_{1113 \mid 123}$ into a different form which is easier to solve. Hence, the number of bed bases of different types in the combined facies models is calculated using the following nine equations.

47 $$\begin{aligned} n_{11}= & {} n_1V_1(p_{11 \mid 1}+p_{11 \mid 12}p_{1111 \mid 12}+p_{11 \mid 13}p_{1111 \mid 13}+p_{11 \mid 123}p_{1111 \mid 123}), \end{aligned}$$

48 $$\begin{aligned} n_{12}+n_{13}= & {} n_1V_1(p_{11 \mid 12}p_{1112 \mid 12}+p_{11 \mid 13}p_{1113 \mid 13}+p_{11 \mid 123}(1-p_{1111 \mid 123}-p_{11- \mid 123})) \nonumber \\{} & {} +n_1(1-V_1)(p_{10 \mid 2}p_{1012 \mid 2}+p_{10 \mid 3}p_{1013 \mid 3}+p_{10 \mid 23}(1-p_{10- \mid 23})), \end{aligned}$$

49 $$\begin{aligned} n_{10}= & {} n_1(1-V_1)(1-V_2)(1-V_3), \end{aligned}$$

50 $$\begin{aligned} n_{22}= & {} n_2V_2(p_{22 \mid 2}+p_{22 \mid 12}p_{2222 \mid 12}+p_{22 \mid 23}p_{2222 \mid 23}+p_{22 \mid 123}p_{2222 \mid 123}), \end{aligned}$$

51 $$\begin{aligned} n_{21}+n_{23}= & {} n_2V_2(p_{22 \mid 12}p_{2221 \mid 12}+p_{22 \mid 23}p_{2223 \mid 23}+p_{22 \mid 123}(1-p_{2222 \mid 123}-p_{22- \mid 123})) \nonumber \\{} & {} +n_2(1-V_2)(p_{20 \mid 1}p_{2021 \mid 1}+p_{20 \mid 3}p_{2023 \mid 3}+p_{20 \mid 13}(1-p_{20- \mid 13})), \end{aligned}$$

52 $$\begin{aligned} n_{20}= & {} n_2(1-V_1)(1-V_2)(1-V_3), \end{aligned}$$

53 $$\begin{aligned} n_{33}= & {} n_3V_3(p_{33 \mid 3}+p_{33 \mid 13}p_{3333 \mid 13}+p_{33 \mid 23}p_{3333 \mid 23}+p_{33 \mid 123}p_{3333 \mid 123}), \end{aligned}$$

54 $$\begin{aligned} n_{31}+n_{32}= & {} n_3V_3(p_{33 \mid 13}p_{3331 \mid 13}+p_{33 \mid 23}p_{3332 \mid 23}+p_{33 \mid 123}(1-p_{3333 \mid 123}-p_{33- \mid 123})) \nonumber \\{} & {} +n_3(1-V_3)(p_{30 \mid 1}p_{3031 \mid 1}+p_{30 \mid 2}p_{3032 \mid 2}+p_{30 \mid 12}), \end{aligned}$$

55 $$\begin{aligned} n_{30}= & {} n_3(1-V_1)(1-V_2)(1-V_3). \end{aligned}$$

These equations contain significantly fewer surface outcome probabilities than are listed in Table 2 for F1, and in the equivalent lists for F2 and F3. Many of those that remain define particular cases of a common general condition. For example, $P_{2222 \mid 12}$ and $P_{3333 \mid 23}$ both represent the probability that a thicker bed (an F2 or F3 bed, respectively) is preserved in the merged models when the amalgamation surface overlaps a bed from a thinner facies (F1 or F2, respectively). Hence, both probabilities can be expressed using the general term $P_{BBBB \mid AB}$ where A and B can be any of facies 1, 2 or 3, provided $T_B \ge T_A$. Note that this term, and the other similar ones discussed below, use A and B as general facies identifiers rather than X and Y which are used elsewhere. This is to make clear that facies X and Y can be any of facies 1, 2 or 3, but facies A and B are constrained by the requirement that $T_B \ge T_A$. The various surface outcome probabilities are calculated in the following sub-sections.

#### Outcomes for Amalgamated Bed Bases in the Presence of One Other Facies

The first set of conditions considered is when an amalgamated bed base in the single-facies model overlaps with a bed of a different facies in one of the other single-facies models. There are four basic groups of situation for this, which have all been discussed already for the two-facies models in Sect. 4.

Preservation of an amalgamation to a thinner bed in the presence of a thicker bed ($p_{AAAA \mid AB}$) has been calculated for the specific case of $p_{1111 \mid 12}$ (Eq. 26), but also applies to $p_{1111 \mid 13}$ and $p_{2222 \mid 23}$. The general form is

56 $$\begin{aligned} p_{AAAA\mid AB}= \frac{n_A(1-V_A)(1-V_B)}{V_AV_B}\Bigg ( \frac{e^{(n_A+n_B)T_A}-1}{n_A+n_B}- \frac{e^{n_AT_A}-1}{n_A} \Bigg ). \end{aligned}$$

Probabilities $p_{1112 \mid 12}$, $p_{1113 \mid 23}$ and $p_{2223 \mid 23}$ refer to the case where a bed from a thinner facies beneath an amalgamation surface is replaced by a bed from a thicker facies. They are of the form given by Eq. 27 as

57 $$\begin{aligned} p_{AAAB\mid AB}= \frac{n_A(1-V_A)(1-V_B)}{V_AV_B}\Bigg ( \frac{e^{(n_A+n_B)T_A}-e^{n_BT_A}}{n_A}- \frac{e^{(n_A+n_B)T_A}-1}{n_A+n_B} \Bigg ).\nonumber \\ \end{aligned}$$

Probabilities $p_{2221 \mid 12}$, $p_{33331 \mid 13}$ and $p_{3332 \mid 23}$ refer to cases where a bed from a thicker facies beneath an amalgamation surface is replaced by a bed from a thinner facies. $p_{2221 \mid 12}$ is given in Eq. 31, and the general form is

58 $$\begin{aligned} p_{BBBA\mid AB}= \frac{n_B(1-V_A)(1-V_B)}{V_AV_B}\Bigg ( \frac{e^{(n_A+n_B)T_A}-e^{n_AT_A}}{n_B}- \frac{e^{(n_A+n_B)T_A}-1}{n_A+n_B} \Bigg ). \end{aligned}$$

Finally, $p_{BBBB \mid AB}$ refers to the case where the thicker bed beneath an amalgamation surface is preserved in the presence of a bed from a thinner facies. Since it is impossible for a thinner bed to erode out the base of a thicker bed (i.e. $p_{BB- \mid AB}=0$), we have

59 $$\begin{aligned} p_{BBBB\mid AB}= 1-p_{BBBA\mid AB}. \end{aligned}$$

#### Outcomes for Non-Amalgamated Bed Bases in the Presence of One Other Facies

Equations 47 to 55 contain two forms of outcome for the case where a non-amalgamated bed base in the single-facies model is converted to an amalgamated base in the presence of one other facies. Probability $p_{1012 \mid 2}$ was derived in the two-facies case (Eq. 29), and $p_{1013 \mid 3}$ and $p_{2023 \mid 3}$ are of the same form. The general expression for these is

60 $$\begin{aligned} p_{A0AB\mid B}= \frac{(1-V_B)}{V_B} ({e^{n_BT_A}-1}). \end{aligned}$$

In general, $p_{A0AB \mid B}<1$, since it is possible for the thicker FB bed to entirely erode out the thinner FA bed above the surface (i.e. $p_{A0- \mid B}>0$).

The other form refers to the case resulting in a bed from a thicker facies overlying a thinner one ($p_{B0BA \mid A}$). In this case, $p_{B0- \mid A}=0$, and therefore

61 $$\begin{aligned} p_{B0BA\mid A}=1. \end{aligned}$$

#### Outcomes for Bed Bases in the Presence of Two Other Facies

The final eight surface outcome probabilities in Eqs. 47 to 55 are not of a form that has already been considered for the two-facies case, since they involve interactions between beds of all three facies. They can, however, be calculated using similar considerations to those applied for the two-facies cases (Figs. 6, 7, Sect. 4.3), and therefore their derivations are not given, and only the final equations are reported here. Unlike those described above, these probabilities cannot be expressed in general forms since the relative thicknesses of all three beds are important. However, they can be discussed in three related groups.

The first group considers the probabilities that unamalgamated bed bases in the single-facies model are eroded when they overlie locations occupied by beds in both other single-facies models. These probabilities are

62 $$\begin{aligned} p_{10-\mid 23}= & {} \frac{(1-V_2)(e^{n_2T_2}-e^{n_2T_1})}{V_2}\nonumber \\{} & {} +\Bigg (1- \frac{(1-V_2)(e^{n_2T_2}-e^{n_2T_1}}{V_2}\Bigg ) \frac{(1-V_3)(e^{n_3T_3}-e^{n_3T_1})}{V_3}, \end{aligned}$$

and

63 $$\begin{aligned} p_{20-\mid 13}= 1- \frac{(1-V_2)(e^{n_2T_1}-1)}{V_2}. \end{aligned}$$

Although $p_{10- \mid 23}$ and $p_{20- \mid 13}$ express similar conditions, the latter is a simpler equation since only one facies (F3) can erode the F20 surface, but the F10 surface can be eroded by both F2 and F3. Since neither F1 nor F2 can erode out an F30 surface, $p_{30- \mid 12} = 0$ and therefore does not appear in Eq. 54.

The second group of probabilities define conditions where amalgamated bed bases in the single-facies models are preserved where they overlie locations occupied by beds in both other single-facies models. It turns out that for F1 and F2, these surface outcome probabilities are the same as the facies preservation probabilities given in Eqs. 39 and 40. Hence,

64 $$\begin{aligned} p_{1111\mid 123}= p_{1\mid 123} \end{aligned}$$

and

65 $$\begin{aligned} p_{2222\mid 123}= p_{2\mid 123}. \end{aligned}$$

It follows that

66 $$\begin{aligned} p_{3333\mid 123}= 1-p_{1\mid 123}-p_{2\mid 123}. \end{aligned}$$

The final group of probabilities assesses whether amalgamated bed bases in the single-facies models are eroded out of the system. These turn out to be the same as the probabilities of erosion of the equivalent unamalgamated surfaces (Eqs. 62 and 63). Hence, $p_{33-\mid 123}=0$,

67 $$\begin{aligned} p_{11-\mid 123}= p_{10-\mid 23} \end{aligned}$$

and

68 $$\begin{aligned} p_{22-\mid 123}= p_{20-\mid 13}. \end{aligned}$$

### Verification of the Equations

**Fig. 10:**
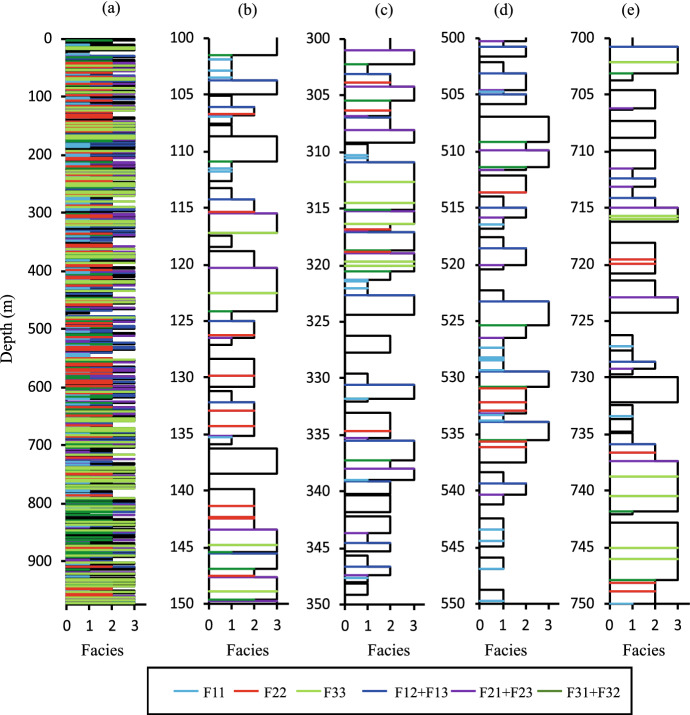
**a** A full-length realisation of the median model of the thee-facies experiment and **b**–**e** four 100 m thick sub-samples of the realisation

**Fig. 11:**
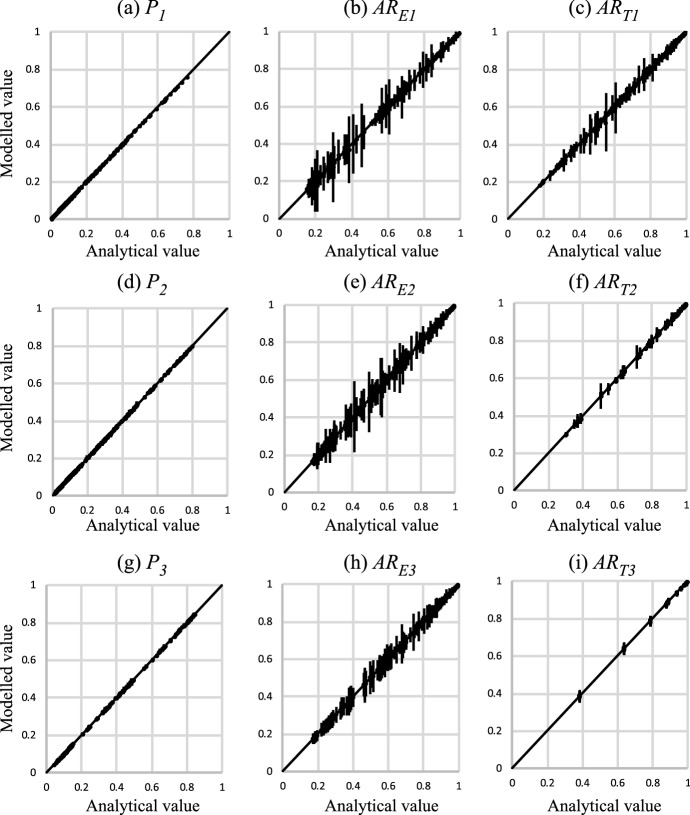
Comparison between the analytically derived facies proportions and amalgamation ratios, and those obtained in a numerical model, for the three facies cases. Error bars represent +/- one standard deviation around the mean value obtained in 100 realisations. See text for discussion

Equations 1, 2 and 34–68 provide an analytical solution for the stacking statistics of models containing three foreground facies. The results are verified in the same way as for the two-facies results (Sect. 4.4), by conducting a high-resolution numerical experiment and comparing the stacking properties obtained in numerical models with those calculated using the equations. In this case, 100 realisations of 243 (i.e. $$3^5$$) models are examined, comprising all possible combinations of three settings of five variables (Table 3). The models are higher resolution than those used for the two-facies cases, since when three facies are present, each bed can create a wider variety of surface types which sometimes have only a very low probability of occurring. Hence, model height (*H*) is defined on the basis that at least 300 beds are expected for each facies. The median model ($$V_1=V_2=V_3 = 0.5$$, $$ T_2$$ = 1.5 m, $$T_3$$ = 2.25 m, $$H = 974$$ m) is shown as an example (Fig. 10). Different positions are shown from this single realisation, highlighting the wide variability in stacking patterns and frequencies of bed base types which can be present within logs that sample only a few tens of each bed type. Even with samples of at least 300 beds of each facies, there is still quite a wide range of values obtained in the experiment, particularly for $$AR_E$$ (Fig. 11b, e, h), as reflected by the length of the error bars. However, the scatter is unbiased, and all parameters are estimated accurately by the equations. Therefore, we conclude from this comprehensive experiment that the analytical expressions for facies proportions and amalgamation ratios are robust.

**Table 3:** Parameters used to verify the three-facies models. All systems have $T_1$ = 1 m

Variable	Value
$V_1$	[0.15, 0.5, 0.85]
$V_2$	[0.15, 0.5, 0.85]
$V_3$	[0.15, 0.5, 0.85]
$T_2$	[1.0 m, 1.5 m, 5.0 m]
$T_3/T_2$	[1.0, 1.5, 5.0]

## Application of the Results

The principal results of this paper are the equations defining the stacking statistics of systems containing two or three foreground facies, presented in Sects. 4 and 5. The reason for deriving these equations is to provide a firm analytical foundation for applying the compression method to facies models containing several foreground facies. The compression method allows object-based facies models to be built in which amalgamation ratio and facies proportions are defined independently and honoured in the output model (e.g. Walsh and Manzocchi 2021a, b). The compression method in general, and the specific results derived in this paper, are applicable to either high or low net:gross systems. However, the approach is particularly relevant for geological systems in which readily available facies modelling approaches are unable to create models with geologically realistic amalgamation ratios (Manzocchi et al. in press b). An example of such a system is deep-water turbidite deposits in which coupled deposition of shale drapes over sand lobes can result in systems with low AR at high NTG.

The compression algorithm is a two-step process. In step 1 a conventional facies model is created with initial facies proportions and object thicknesses ($PI_X$ and $TI_X$, respectively) set to values that will ensure the model has the target amalgamation ratios. In step 2, the thicknesses of model cells are expanded by facies-specific expansion factors ($E_X$ for the foreground facies and $E_0$ for the background facies) which transform the initial thicknesses and facies proportions to their target values without changing the amalgamation ratios. The result is a model with independently defined $AR_X$, $P_X$ and $T_X$ values.

If a model contains only one foreground facies (F1), the facies proportion ($P_X$) is equal to the net:gross ratio (*NTG*) by definition. There is only one definition of *AR* in such a model, and if all beds are the same thickness, *AR* is linked to *NTG* through the expression

69 $$\begin{aligned} AR=NTG. \end{aligned}$$

This relationship, which is also derived in the present paper but expressed using different symbols (Eq. 6), provides the basis of the geometrical transformation underlying the compression algorithm when applied to a model containing a single foreground facies (Manzocchi et al. 2007; Walsh and Manzocchi 2021a).

The single-facies algorithm is parameterised using a compression factor ($c_F$) which represents the ratio between the amount by which the thickness of shale and sandstone cells must be modified during the geometrical transformation associated with the compression algorithm (Manzocchi et al. 2007). Hence,

70 $$\begin{aligned} c_F=E_0/E_1. \end{aligned}$$

AR is linked to NTG through

71 $$\begin{aligned} c_F=\frac{1-{1/NTG}}{1-{1/AR}}. \end{aligned}$$

If $c_F=1$, then $AR=NTG$, which, as discussed, is the case in a constant-thickness object-based model. However, natural depositional systems often have $c_F<<1$, with values of 0.05 to 0.3 typical (e.g. Manzocchi et al. 2007, 2020; Romans et al. 2009; Zhang et al. 2015; Pyles et al. 2019; Mueller et al. 2017; Soni et al. 2020). In these cases, application of the compression method using $PI_1 = AR$ in step 1 would result in a geometrically transformed object-based model with the correct stacking characteristics.

The simple relationship (Eq. 69) arises because bed bases cannot be eroded in models of constant-thickness beds. Therefore, the equivalent relationship for multi-facies models applies only to the thickest facies present (F3 in the three-facies models), since the thinner F1 or F2 bed bases can be eroded, which will influence their amalgamation statistics. Since all F3 bed bases are preserved in the model, the probability that one of them overlies any one of F1, F2 or F3 is given by the overall net:gross ratio. This means that

72 $$\begin{aligned} AR_{T3}=P_1+P_2+P_3, \end{aligned}$$

which has been verified by examining the results of the experiment described in Sect. 5.3. However, no similar, simple relationships exist for $AR_{T1}$ and $AR_{T2}$. Therefore, although it is possible to create multi-facies compression-based models which honour user-defined input values of $AR_{TX}$ (see Manzocchi et al. in press b for an example with six facies distributed in pairs over two hierarchical levels), it is much simpler to use the alternative definition of amalgamation ratio ($AR_{EX}$) when parameterising systems for use in compression-based modelling. This approach is developed below.

Evaluation of stacking property values calculated from the equations for multi-facies models shows that the effective amalgamation ratio ($AR_{EX}$) is equal to an effective facies proportion ($P_{EX}$) which is defined on the basis of only the foreground facies in question and the background facies. Hence,

73 $$\begin{aligned} P_{EX}= & {} AR_{EX}, \end{aligned}$$

74 $$\begin{aligned} \text { where } P_{EX}= & {} \frac{P_X}{P_X+P_0}. \end{aligned}$$

**Fig. 12:**
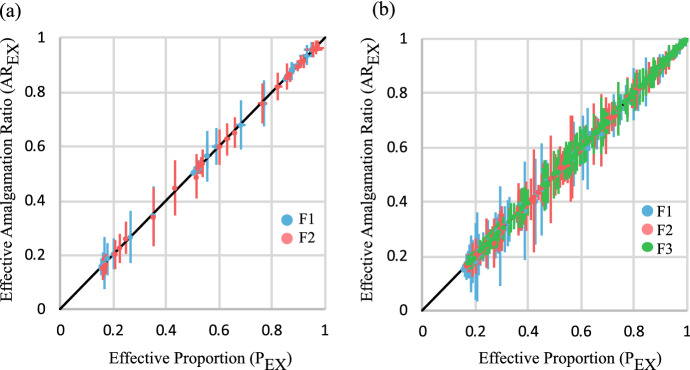
Cross-plots of $AR_{EX}$ versus $P_{EX}$ for **a** the two-facies models and **b** the three-facies models

This relationship is confirmed by the results of the two- and three-facies experiments (Fig. 12) and is compatible with the known single-facies relationship (Eq. 69), since in that case, $P_{EX} = P_X=NTG$.

Equation 73 provides the anchor needed to apply the compression method to multiple-facies models. In multiple-facies models, facies-specific compression factors ($c_{FX}$) are related to $P_{EX}$ and $AR_{EX}$ through the expression

75 $$\begin{aligned} c_{FX}=\frac{1-{1/P_{EX}}}{1-{1/AR_{EX}}}. \end{aligned}$$

**Fig. 13:**
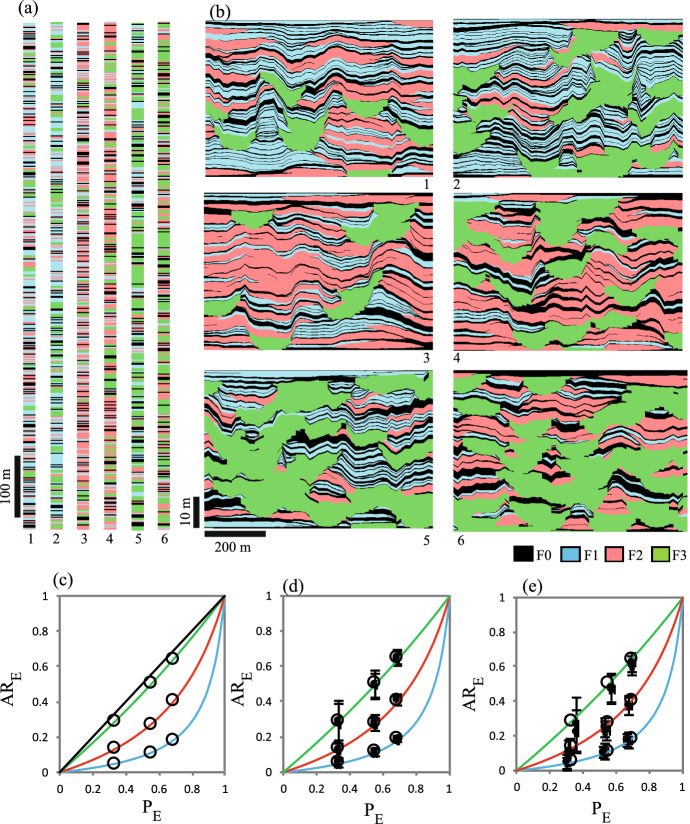
Example three-facies models built using the compression method and parameterised using the new results. **a** Continuum one-dimensional models. **b** Gridded two-dimensional models. The plots of $AR_E$ versus $P_E$ show the target properties of the three settings used for each facies **c**, and the resultant properties obtained in multiple realisations of **d** the 1D and **e** the 2D models. Error bars show +/- one standard deviation of properties derived from 100 realisations of each case. The coloured curves in c to e are curves of $c_F$ = 0.1, 0.3 and 0.8, and the black line in c shows $c_F$ = 1.0. See text for discussion

Figure 13a and b shows one- and two-dimensional compression-based models for facies with $c_{F1}=0.1$ (blue facies), $c_{F2}=0.3$ (pink facies) and $c_{F3}=0.8$ (green facies). Six cases are considered, representing the different possible combinations of $P_X$ = 0.1, 0.25 and 0.45 (Table 4). In each case, $AR_{EX}$ has been calculated as a function of $P_{EX}$ and $c_{FX}$ using Eq. 75: the combinations of $P_{EX}$ and $AR_{EX}$ values defining each facies are shown overlying the corresponding $c_{FX}$ curves in Fig. 13c.

Although $c_{FX}$ is a very useful parameter for understanding the geometry of compression-based facies models and relating them to natural systems, it is not used directly in the compression algorithm which instead uses facies-specific expansion factors ($E_X$, see Walsh and Manzocchi 2021a for details). The four $E_X$ values can be derived as a function of the three $c_{FX}$ and $P_X$ values (Table 4) by solving the equations

76 $$\begin{aligned} {E_0}/{E_1}= & {} c_{F1}, \end{aligned}$$

77 $$\begin{aligned} {E_0}/{E_2}= & {} c_{F2}, \end{aligned}$$

78 $$\begin{aligned} {E_0}/{E_3}= & {} c_{F3}, \end{aligned}$$

and

79 $$\begin{aligned} \frac{1-P_1-P_2-P_3}{E_0}+\frac{P_1}{E_1}+\frac{P_2}{E_2}+\frac{P_3}{E_3}=1. \end{aligned}$$

Once the expansion factors are determined, the thicknesses of each facies, and the facies proportions in the initial step 1 model ($TI_X$ and $PI_X$), can be determined from the expressions

80 $$\begin{aligned} TI_X=T_X/E_X, \end{aligned}$$

and

81 $$\begin{aligned} PI_X=P_X/E_X. \end{aligned}$$

Although the target thickness value for each facies is identical in all six cases, they all have different values of $TI_X$ (Table 4), illustrating the dependency of $E_X$ on the properties of all the facies in the model (Eqs. 76 to 79).

**Table 4:** Parameters used in the six compression-based cases examined in Fig. 13

		Case 1	Case 2	Case 3	Case 4	Case 5	Case 6
Target properties	$P_1$	0.45	0.45	0.25	0.1	0.25	0.1
	$P_2$	0.25	0.1	0.45	0.45	0.1	0.25
	$P_3$	0.1	0.25	0.1	0.25	0.45	0.45
	$AR_{E1}$	0.1837	0.1837	0.1111	0.0476	0.1111	0.0476
	$AR_{E2}$	0.2727	0.1304	0.403	0.403	0.1304	0.2727
	$AR_{E3}$	0.2857	0.5	0.2857	0.5	0.6429	0.6429
Expansion factors	$E_0$	0.4	0.475	0.44	0.545	0.615	0.645
	$E_1$	4	4.75	4.4	5.45	6.15	6.45
	$E_2$	1.3333	1.5833	1.4667	1.8167	2.05	2.15
	$E_3$	0.5	0.5937	0.55	0.6812	0.7687	0.8063
Input properties for intial facies modelling	$V_1$	0.1798	0.1812	0.107	0.0455	0.109	0.0461
	$V_2$	0.2345	0.1067	0.3739	0.3834	0.1117	0.2514
	$V_3$	0.2037	0.4244	0.1871	0.3765	0.5891	0.5658
	$T_1$	0.25	0.2105	0.2273	0.1835	0.1626	0.155
	$T_2$	1.5	1.2632	1.3636	1.1009	0.9756	0.9302
	$T_3$	10	8.4211	9.0909	7.3394	6.5041	6.2016
	$PI_1$	0.1125	0.0947	0.0568	0.0183	0.0407	0.0155
	$PI_{2}$	0.1875	0.0632	0.3068	0.2477	0.0488	0.1163
	$PI_{3}$	0.2	0.4211	0.1818	0.367	0.5854	0.5581

The target facies thickness ($T_1$= 1 m, $T_2$ = 2 m, $T_3$= 5 m) and compression factors ($c_{F1}= 0.1$, $c_{F2}= 0.3$, $c_{F3}= 0.8$) are the same for all six cases. The one-dimensional models have *H* = 1000 m, and the two-dimensional models have *H* = 50 m, width (*W*) = 1000 m, with individual objects having $W_1$ = 800 m, $W_2$= 400 m and $W_3$ = 200 m. These dimensions are arbitrary, and the systems are not based on any specific example

Facies proportions are the user-defined input required by many object-based modelling software packages, in which case Eqs. 80 and 81 provide all the information needed to construct the step 1 model. However, the object-based models used in this paper are built from facies-independent volume fractions ($V_X$) rather than from merged facies proportions, and therefore, an extra step is required in which Eqs. 34 to 36 are used to determine the initial volume fractions ($VI_X$) as a function of the initial facies proportions and thicknesses ($PI_X$ and $TI_X$) given by Eqs. 80 and 81. This inversion is done numerically.

Figure 13d compares $PI_X$ and $AR_{EX}$ values obtained in continuum one-dimensional models (examples of which are shown in Fig. 13a) which have a similar resolution to those used previously (e.g. Fig. 10). The mean $PI_X$ and $AR_{EX}$ values match accurately the target values (the large circles) with similar variability in $AR_{EX}$ between realisations as observed previously (e.g. Fig. 11b, c, h). These one-dimensional models therefore validate the approach described above (Eqs. 73 to 81).

The two-dimensional models (Fig. 13b) were constructed using the implementation of compression-based modelling discussed in detail by Manzocchi et al. (in press b). These models are more representative of the model resolution that might be used in practice, and differ from the one-dimensional models in at least three important ways. First, at 50 m thickness, the models are only a few times thicker than the thickest object present ($TI_3$ = 10 m in case 1; Table 4), and therefore any single realisation is unlikely to be representative. Second, unlike the one-dimensional models where objects are placed in a continuum, these two-dimensional models are gridded. The grid cells in these examples were scaled to be six times thinner than the thinnest objects in the initial model, which means that the models have a consistent resolution, but the presence of a grid will introduce a systematic bias in facies connectivity and hence amalgamation ratios (e.g. Koza et al. 2014). Third, the objects have tapered shapes and terminate laterally within the model area. This leads to the systematic bias in $P_X$ of compression models discussed by Walsh and Manzocchi (2021a), who proposed and applied a third step in the algorithm to correct for it (this step has not been applied here). These biases in $PI_X$ and $AR_{EX}$ account for the small differences between the target values and the mean values obtained in 100 realisations of the two-dimensional models, which nonetheless show a reasonably close correspondence between target and model stacking characteristics (Fig. 13e). Hence, the new expressions for amalgamation ratios and facies proportions derived in this paper provide the analytical foundations for applying the compression method to multi-facies object-based models.

## Summary and Conclusions

This paper has developed equations for the expected facies proportions and amalgamation ratios of object-based models consisting of one, two or three foreground facies of constant-thickness beds embedded in an additional background facies. Expressions have been derived for the probabilities of facies preservation given a stratigraphically meaningful stacking of beds, and for the total numbers of bed bases which are unamalgamated or have different types of amalgamation surface. The results have been validated by comparing their predictions against the same statistics measured in multiple realisations of large, one-dimensional models built using the same assumptions that were made in the analytical solutions.

The objective of the work has been to provide a firm theoretical basis for applying the compression-based modelling approach to systems containing multiple foreground facies. The most important result for this is the demonstration that $P_{EX}=AR_{EX}$. This relationship underlies a relatively simple procedure for determining the parameters required to construct compression-based models including multiple facies parameterised interpedently. The procedure has been illustrated using a set of well-parameterised two-dimensional compression-based models containing a background and three foreground facies. Although the paper has only considered cases with one, two and three foreground facies, it is likely that $P_{EX}=AR_{EX}$ irrespective of the number of facies present, and therefore that the procedure developed can be applied more generally.

## Appendix

A summary and explanation of the terminology used is provided here. Certain symbols introduced on the fly and used only at specific points of the derivation (e.g. Sect. 4.2) are not included in this list.

- Background facies: F0
- Foreground facies: FX, FY and (occasionally) FW are used as general terms to refer to any of the three foreground facies considered. F1, F2 and F3 are specifically named to satisfy the conditions that $T_1 \le T_2 \le T_3$ which is fundamental to the equations. FA and FB are used in some of the equations to refer to any two facies for which $T_A \le T_B$.
- Unamalgamated bed base: FX0 signifies that a bed of facies X lies over the background facies.
- Amalgamated bed base: FXY signifies that the base of a bed of facies X is in contact with a bed of facies Y.
- Number of beds in the single-facies stacked FX model: $n_X$
- Number of FXY bed bases in the single or combined stacked model: $n_{XY}$
- Total amalgamation ratio: $AR_{TX}$. This applies to a foreground facies.
- Effective amalgamation ratio: $AR_{EX}$. This applies to a foreground facies.
- Model height (m): *H*.
- Bed thickness (m): $T_X$. This is specific for individual foreground facies.
- Bed thickness in the single-facies stacked model (m): $t_X$. This is $\le T_X$, since the bed may or may not be partially eroded.
- Facies volume fraction: $V_X$.
- Facies proportion in the combined stacked model: $P_X$.
- Effective facies proportion in the combined stacked model: $P_{EX}$.
- Facies transition probability: $p_{XY}$. The probability that facies X transitions downwards into facies Y.
- Facies preservation probability: $p_{X \mid XY}$, $p_{X \mid XYW}$. This defines the probability that the facies in the single-facies model is preserved in the combined stack model given an overlap with one or more other facies. The single character to the left of $\mid$ indicates which facies is preserved. The two or three characters to the right of $\mid$ are a list of the facies present at this location across the full set of single-facies stacked models.
- Surface overlap probability. This defines the probability that a bed base in the single-facies model is in the same location as beds of other facies. The general term used for this is $p_{OA}$, with specific probabilities having forms like $p_{XX \mid XYW}$ or $p_{X0 \mid Y}$. The two characters to the left of $\mid$ indicate a surface type in the single-facies stacked model (FXX and FX0 in these examples), and the one, two or three characters to the right of $\mid$ list the facies that are present in the other single-facies stacked models directly beneath this surface.
- Surface outcome probability. This defines the probability of a specific outcome for a surface in the single-facies model, if a specific overlap is present. Outcomes include preservation of the surface, transformation of it into a different type of surface, or erosion of the surface out of the system. The general term used for this is $p_{OB}$, and specific probabilities have forms like $p_{XXXY \mid XYW}$ or $p_{X0- \mid Y}$. There are either four characters to the left of $\mid$, or two characters followed by “-”. The first two characters indicate a surface type in the single-facies stacked model. The third and fourth characters indicate the outcome surface type in the combined model. Replacement of these by “-” indicates that the surface is eroded out and does not exist in the combined model. The one, two or three characters to the right of $\mid$ list the facies that are present in the single-facies stacked models directly beneath this surface.
- Initial bed thickness in step 1 of the compression algorithm (m): $TI_X$.
- Initial facies volume fraction in step 1 of the compression algorithm: $VI_X$.
- Initial facies proportion in step 1 of the compression algorithm: $PI_X$.
- Compression factor: $c_{FX}$. This is facies-specific for all foreground facies.
- Expansion factor: $E_X$. This is facies-specific for all facies (foreground and background).
